# Developing nucleic acid-based electrical detection systems

**DOI:** 10.1186/1475-2859-5-9

**Published:** 2006-03-02

**Authors:** Magdalena Gabig-Ciminska

**Affiliations:** 1 School of Biotechnology, Royal Institute of Technology KTH, S-10691 Stockholm, Sweden; 2 Laboratory of Molecular Biology (affiliated with the University of Gdansk), Institute of Biochemistry and Biophysics, Polish Academy of Sciences, PL-80822 Gdansk, Poland

## Abstract

Development of nucleic acid-based detection systems is the main focus of many research groups and high technology companies. The enormous work done in this field is particularly due to the broad versatility and variety of these sensing devices. From optical to electrical systems, from label-dependent to label-free approaches, from single to multi-analyte and array formats, this wide range of possibilities makes the research field very diversified and competitive. New challenges and requirements for an ideal detector suitable for nucleic acid analysis include high sensitivity and high specificity protocol that can be completed in a relatively short time offering at the same time low detection limit. Moreover, systems that can be miniaturized and automated present a significant advantage over conventional technology, especially if detection is needed in the field. Electrical system technology for nucleic acid-based detection is an enabling mode for making miniaturized to micro- and nanometer scale bio-monitoring devices via the fusion of modern micro- and nanofabrication technology and molecular biotechnology. The electrical biosensors that rely on the conversion of the Watson-Crick base-pair recognition event into a useful electrical signal are advancing rapidly, and recently are receiving much attention as a valuable tool for microbial pathogen detection. Pathogens may pose a serious threat to humans, animal and plants, thus their detection and analysis is a significant element of public health. Although different conventional methods for detection of pathogenic microorganisms and their toxins exist and are currently being applied, improvements of molecular-based detection methodologies have changed these traditional detection techniques and introduced a new era of rapid, miniaturized and automated electrical chip detection technologies into pathogen identification sector. In this review some developments and current directions in nucleic acid-based electrical detection are discussed.

## Review

### Introduction

Biological macromolecules, such as nucleic acids, both deoxyribonucleic acid (DNA) and ribonucleic acid (RNA), and their analysis had an essential role in the rapid development of molecular genomics, biotechnology and medical diagnostics. One of the fastest growing areas in DNA/RNA analysis was the development of nucleic acid-based chips. Since their first development in the late 80s, nucleic acid chips have evolved into an important tool providing complex informative data. During the last years, this field quickly branched out, however, in its methodology and application. Different names for the chips, such as DNA/RNA chips, Biochips, Genechips, Biosensors or DNA arrays appeared [1,2]. Generally, a biochip is known as plastic, glass or silicon wafer that may rapidly detect chemical agents from biological material [3]. In the detections used most routinely in the area of chip technology, the participation of reporter molecules is observed. This type of sensors assures the high sensitivities, although paying greater attention label-independent electrical detection systems are even more promising, especially for application where for instance the analysis duration is more critical than the detection limit, or where the direct real time monitoring is desired [4-8]. Also, by eliminating the labeling steps from the protocol, simplicity of readout and increase in speed is obtained. Even though the development of such chips is still in its infancy, the detection of very low level, i.e. few molecules is expected.

In this review, the progress in electric chip operation, for both indirect detection of labeled molecules and direct analysis of label-free nucleic acids, is discussed mostly in respect to pathogen detection, as nucleic acid chip technology is receiving much interest as a potent tool for pathogenic microorganism detection [9-13]. Most likely, the detection of pathogens is nowadays somehow more important than it has been in the past. One serious problem is the threat of bioweapon attacks by terrorist organizations with microorganisms like *Bacillus anthracis *(anthrax), but also appearance of rapidly evolving pathogens, causing severe diseases, like SARS (by *Coronavirus*) and bird flu epidemics (by *Orthomyxovirus*) as well as antibiotic-resistant microorganisms. Moreover, food contamination by pathogenic microorganisms has made the development of fast, reliable, and sensitive analytical methods for use in monitoring of pathogens very important [12,14,15]. Methods for routine identification of microorganisms take at least several hours, a day or even more. Typically they take between 24~48 hrs to yield results. They rely on time-consuming growth in culture media, followed by isolation, biochemical identification, and sometimes serological determination [16-18]. The culture-based tests have a relatively low sensitivity, about 30~50%. That means for example that the probability of identifying a particular bacterium from the sepsis patient is less than 50% [18]. That is where new analytical techniques offer significant benefits. The developments in bioinformatics have widened the basis for organism identification to include also nucleic acid analysis. As a result, new analytical instruments, monitoring devices and rapid test kits have been created to detect and quantify bacteria [10,19-21]. Among them, real-time PCR systems are playing an increasingly important role. Although, very sensitive and suitable for routine use in analytical laboratories, they remain however limited for in field applications because of their inherent complexity. In this respect, most promising breakthrough in the area of rapid bacterial detection turned into biochip technology. Automated nucleic acid sensing biochip systems became a great tool for detection of different toxic microorganisms [8,12]. Their potential to be quantitative, useful even for unculturable microorganisms and lack of technical barriers for their deployment in the field cannot be overlooked.

### From optical to electrical systems, and from label-dependent to label-free approaches

Chip structure creates various advantages [2]. Users of this unique platform for nucleic acid analysis realize significant profits compared to other available conventional detection techniques.

**Figure 1:**
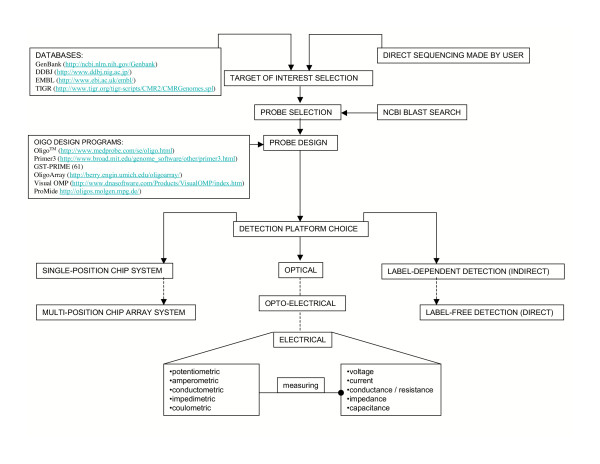
Directions and strategies in the field of nucleic acid-based chip detection.

Apart from the underlying technology, biochips can be discerned by their evaluation method, which may be optical or electric (Figure 1) [22]. Most of the biochips available on the market are based on external or internal optical detection (fluorescence or chemiluminescence) [23]. The optical biosensors are also the best studied, but the detection limits are expected to be better for the electrical sensors along with the most simple instrumentation [24,25]. Current optical-based techniques still require a relatively large amount of power, need a bulky chip reader (optics, lasers and cameras), and thus are less applicable to miniaturization [22,25]. In today's optical techniques that often rely on the detection of fluorescence, unknown gene sequences are loaded onto a biochip after first having been tagged with a fluorescent dye emitting light of a particular wavelength when irradiated. A specific optical filter charge-coupled-camera (CCD) or Photo Multiplier Tube (PMT) with high precision mechanical set-up reads the light patterns emitted. These light patterns explain the composition of the substance detected. It should be mentioned that fluorescent dyes used as the standard labels for this type of optical chips are unfortunately very expensive and they can rapidly photo-bleach (the dye is photochemically converted to a non-fluorescent compound). An alternative to the fluorescence detection used in many systems is chemiluminescence format, which overcomes the use of fluorescent dyes used in the first case [23,25,26]. Parallel, much effort and work is continually performed on instrumentation improvements to be incorporated in optical systems, making them more and more feasible.

However, despite the performance of new generation readers with lower cost and diminished foot-print [27], optical readers remain expensive and not portable. This limits their use as a routine tool and also for dispersed testing and typing in the field [22]. The invention here is a biochip system that electronically detects biomolecules. However, it has to be mentioned that the use of an electrical signal rather than the optical signal is over 40 years old with the first experiments in this area being presented by Palecek [28]. Nowadays attractive systems have been described to make the electrical readout more sensitive and functional (Figure 1) [6,29]. They use the flow of electric current as a basis of measurement and analysis. The system is based on two major parts. The first part is a disposable sensor chip, and the second is a device for measurement of the electrical signal [30]. Electrical-based methods are advantageous in that they are more amenable to miniaturization [31]. The intensity of the electrical current indicates not only the presents/absence of the analyte in the sample, as it is in the case of optical systems, but also concentration of the tested substance. However, it should to be mentioned that nowadays also the optical methods are becoming quantitative. Optical biosensors correlate changes in the concentration of applied standards to direct changes in the characteristics of light. Afterwards target analyte response is evaluated.

Since nucleic acids do not have intrinsic properties that are functional in direct detection, many of the nucleic acid-based assays, especially optical setups, require a label in their detection method. No consensus exists on the choice of a label for nucleic acid detection. However, basic parameters such as label stability, sensitivity of detection and its convenience should dictate label choice. Electrical modes were developed for detection of both label-free and labeled objects [6,23]. With label-dependent electric chip analysis method, specific enzymes for instance are added to the samples to be detected. These enzymes catalyze the transformation of an inactive substance, which has been added in a separate step, into an electronically active component. This chemical procedure creates an electrical current in the pico- and nanoampere range at the sensor electrodes, which is measured with highly sensitive circuits. Contrary to indirect detection techniques, where labeling is a requirement to translate the hybridization event into a signal, in direct detection techniques, a target molecule or any other object from the system does not need to be labeled. Although label-dependent methods achieve the highest sensitivities, eliminating the labeling steps simplifies the readout, the speed and ease of nucleic acid assays [24,30,33]. In a label-free approach the immobilized probe recognizes a complementary sequence if the target is present in the sample. Next, the transducer converts the biological interaction into a measurable signal, proportional to the degree of hybridization that is to the amount of target molecule in the sample. Label-free strategies reduce analysis times and cost. They are also free from unfavorable effects from the labels, such as its instability and steric hindrances. Thus, in order to meet requirements of powerful nucleic acid-based readers, the label-independent detection mean, in which hybridization of nucleic acids on the chip can be directly related to an electrical signal, appears as the perfect candidate for this challenge. In fact, many efforts have been already made in this direction and different approaches to achieve an electrical readout for a label-free nucleic acid-based chip were presented. For instance, electrical sensor devices based on impedance/capacitance/field-effect measurements are now well established [32-36]. Using microfabricated silicon field-effect sensors, the changes in surface potential can be monitored when nucleic acid hybridizes on the sensor face. This sensor is similar in structure and behavior to a metal/oxide/semiconductor device, which essentially is a capacitor with variable capacitance, depending on the potential applied. With such detection systems, different targets like pathogen microorganisms can be effectively screened for their presence by the examination of specific genomic regions.

Although increased attention has been given recently to label-free chip detection systems causing significant progress in this area, many aspects crucial to specificity or detection limit remain unsolved. Thus, proper applications of these undoubtedly promising devices would still require a solid intellectual input.

### From single to multi-analyte and array detection format

These days the nucleic acid-based chip devices can be more and more often found in an array format. However, chip-based nucleic acid detection principles can still be effectively employed in many other formats than microarrays. For some applications, when an additional separation procedure or a continuous monitoring of analytes is needed, microarrays may not offer the best detection system for bioanalytics. In single detection format of biochips, the use of micro- or nanoparticles, like magnetic beads with a large surface area for biomolecule attachment, results in very sensible and practical target molecule identification [37,38]. Besides using optical properties of nanoparticles [39,40], electrical detection and quantification principles have been successfully adopted for biochip applications [41-43]. Such metal beads consisting of a superparamagnetic central part surrounded by a polymeric functional surface film suitable for attachment of possible sensing agents, present a versatile scaffold for biomolecular recognition and application in electrical biochip technology [11,29,44,45].

As mentioned above, in recent time array chip technology that adapted the multi-analyte approach has become an indispensable tool for different genomic studies. The construction of a multi-detection system on chip enabled capacious (i.e. high throughput analysis), and at the same time very compactable biosensing in an array format. The nucleic acid-based array can be defined as an ordered collection of spots, each containing single defined species of a target molecule. Large sets of nucleic acid probe sequences are immobilized in defined, addressable locations on the surface of a substrate, which is capable of accessing usually enormous amounts of genetic information from biological samples in a single hybridization assay. Each spot represents the equivalent of a conventional analysis performed in a test tube. High-density microarrays are used for analyzing many thousands of nucleic acid sequences simultaneously in a relatively short time, allowing a researcher to measure typically full genome gene expression with a single array, while medium- and low-density array chip demonstrate in most cases the use for clinical diagnoses and personalized medical care. They are particularly important in a typing of microorganisms, and especially in detection of pathogenic bacteria. Application of microarray chips for analysis of microbial pathogens, and especially for simultaneous typing of several harmful bacteria is an important element of public health these days [13,15,46,47]. Of the two approaches: optical and electrical detection platforms described above, the second one has larger impact for bacterial detection in recent times. As discussed before, the precise identification of optical signals implies the use of highly sophisticated and thus expensive devices. Also, the analysis of the results can be problematical and extensive training is required. In many cases, only specialized bioinformatics centers are equipped sufficiently well to analyze the data properly. In contrast, the development of electrical multi-position chip platforms for biological detection enables a parallel analysis of complex samples in an inexpensive and easy way, thus resulting in performance of devices certainly reachable by individual health care units such as hospitals or clinics. This is especially important when taking into account the increase in the number of cases of food poisoning, the spread of antibiotic resistance in health centers, and also the raise in bioterrorism events. Concluding, it is of high probability that the coming time will see much more high-speed detection of microorganisms, and electrical biosensors will most probably involved in it.

### To automation and miniaturization

The need for small analytical devices for nucleic acid-based electrical detection has resulted in the construction of microchips and microarrays that contributed to microfluidic systems [48,49]. The key factor in the successful introduction of the chip-based nucleic acid detection in on-side or point-of-care use is the miniaturization and automation of the system and procedures. It is very important to differentiate between passive biochips or microarrays, dominating today's market, and active biochip or microarray analytical systems with microfluidic capabilities. Typically, active devices are sensors that perform or assist signal transduction. The fluid can be transported inside the channel by applying an external force in the form of a pressure or potential difference. Also, electrokinetic or electroosmotic flow control realize the solution transport over a network of channels towards the chip surface, while in the case of passive chips this is done using a specific design of the geometry of the channel network. Compared with the passive form of biochips relatively popular nowadays, the active form of biochips has taken on the feature of high sensitivity, which shortens the time for sample analysis. This is undoubtedly an irresistible general trend. Up to now, only few active forms of biochips have come to light in the world. Most of the currently demonstrated microfluidic devices follow single or only a few functionalities. There is a notable lack of functional integration, intended as the possibility of combining the analytical steps required to perform a complete analytical protocol. Due to the difficulty of the sample preparation, most existing biochip systems still carry out this initial step off-chip using conventional bench-top methods. However, a biochip platform for fully integrated genetic assays for sample-to-answer nucleic acid analysis was reported by Liu and co-workers [48]. The biochip device consisting of microfluidic mixers, pumps, valves, tubes and microarray sensors, allows performance of all functions including sample preparation, mixing steps, chemical reaction and electrical detection. With the use of such fully integrated biochip device the researchers reported functional detection of pathogenic bacteria from blood samples. Also, Rudi and his co-workers developed methods in the field of nucleic acid-based microbial sensors that cover both the sample preparation and detection approaches [50]. Furthermore, a new generation of an automatic electric chip measuring system for the detection of biological agents was reported lately [49]. In the most recent instrumental version added microfluidics, i.e. a complex network of valves and tubes, permits users to move liquids on and off the chip allowing for performance of multi-step assays. These miniaturized amperometric devices, based on electrical biochips made in silicon-technology, have been constructed for field applications and point of care diagnoses. An advantage of the developed system is the use of a semiconductor technology that avoids any mechanical adjustments of sensing elements, as it is necessary for optical devices.

Nowadays, the advent of not only micro- but also nano-miniaturization presents new challenges for nucleic acid detection. The advantages of miniaturization in bioanalytics are mainly bound to parallelism, reduced reagent consumption, speed and functional integration. As a result of miniaturization, an ultrasensitivity of nano-devices in nucleic acid analysis is expected, or even requested for many reasons at the present time. This includes detection of genetic material from single cell and in many cases single copy gene analysis. Method to achieve single molecule detection limits (i.e., yoctomoles; 10^-23 ^moles) is a new wave in this area. In this regard, recent nanoscale sensors based on nanowires (NWs), nanotubes (NTs), and other nanomaterials have received considerable attention. Both, silicon NW and NT electronic devices that function as ultrasensitive and selective detectors of biological molecules were reported [51-54]. Generally, NWs and NTs have the potential for very high sensitivity detection. The accumulation or depletion of charge carriers, which are caused by binding of a charged biological target macromolecules such as nucleic acids at the NW or NT device surface, can affect the electrical properties of these nanostructures. The changes in the electrical signal are evidence of the presence of specific molecule of interest in a sample solution.

From another point of view, the trend in miniaturization seems to cope with the need of maintaining the statistical significance of the sample. For instance, when testing water supply it is clear that a drop of such sample would hardly have any cell of interest. Of course, possible solutions to these needs (i.e. related to analyte concentration) will appear and exciting developments for the detection systems will certainly be constructed in the future.

### When applying biochip for nucleic acid-based detection

The identification of pathogens with biochips is based on specific probes, in most cases on oligonucleotide probes that target nucleic acid sequences that are only present solely in the organism of interest to the investigator. To determine suitable sets of probes for nucleic acid-based detection intensive computational studies have to be performed. Each probe is designed using a proprietary oligonucleotide design program. Various factors, such as melting point, secondary structures, cytosine/guanine (C/G) content, and sequence specificity and selectivity need to be considered. Referred parameters should be contemplated in respect to potential merging variants for each gene since these factors have an effect on the specificity and sensitivity of hybridization [55]. In more detail, the strategy for the generation of oligonucleotide probe sets is as follows (Figure 1.): First, each sequence of interest is chosen. In most cases, they are obtained directly from varied databases. Sometimes the nucleic acid sequences are achieved through direct sequencing made by users. Second, suitable sequence regions are chosen from gene probe candidates. Additionally, a NCBI BLAST search tool is used for the analysis of the sequences of particular oligonucleotides. This helps to verify the oligonucleotides' design and to visualize their specificities. By alignment with nucleic acid sequences from other bacterial species, some sites of genes are omitted during probe design. It is assumed that checking for cross homologies may eliminate potential cross hybridizations during detection on the chip. In the next step, each gene-specific probe is designed by using the oligonucleotide design programs. A number of oligonucleotide design softwares that may help in probe selection and analysis is available. To avoid possible effects of degradation of the target nucleic acid when using two probes in the detection system (one capture and one detection), these probes should be located in close proximity to each other. In this way, the chosen probes may guarantee the helper effect to each other when approaching the selected target sequences.

A probe that has recently gained an increasing application is peptide nucleic acid (PNA) [8,56,57]. Probes made of peptide nucleic acid, which have very strong affinity for complementary DNA sequence, can further improve the specificity. Such probes can more effectively discriminate the pathogenic organisms at the level of single-base mismatches. Additionally, PNA/DNA hybrids are resistant to nuclease attack, due to inability of nucleases and proteases to recognize the peptide backbone, have higher thermal stability and their melting temperature is higher than the corresponding DNA/DNA duplex. They are relatively insensitive to ionic strength due to the neutral charge of PNA.

Once the appropriate oligonucleotide probes are chosen, the immobilization of target specific captures onto a solid substrate can be performed. The probes may be deposited either directly on the chip surface or on supporting materials such as microscopic beads. Several effects have to be taken into account with probe immobilization [58]. Thus, it is important that the immobilization chemistry is stable during subsequent assay steps and that the probes have to be functional after attachment. The probes must be immobilized with an appropriate orientation and configuration so that base pairing is not restrained. There should not exist steric impediments or lack of accessibility due to the dense packing of the immobilized probes. Finally, it is necessary to characterize the immobilization efficiency and immobilized probe functionality.

When applying nucleic acid-based detection system it is very important to select proper gene sequences as targets. Generally, DNA provides evidence for the presence/absence of microorganisms, rRNA is an indicator of cell activity or viability, and mRNA provides evidence for specific activity and expression of functions. In a cell, DNA is present only in very low amounts, usually just as a single copy per cell. Thus, one strategy to overcome the problem with sensitivity limits of the biochip when analyzing DNA it is to target ribosomal RNA that is present in bacteria cells at a high copy number (for *E. coli *around 20 000 per cell). The detection based on mRNA may also be considered.

Various types of nucleic acid targets have been selected for detection of pathogens, however due to the sensitivity limitations in most cases their artificial analogs such as PCR products were used directly in the assays for biochip measurements. To date, only a handful of scientists worldwide have reported electrical detection of nucleic acids isolated from cells [[11,13,43,45], ]. They have demonstrated that electrochemistry can be used to detect nucleic acids directly from cell homogenates. For instance, in the absence of a PCR step it has been found that 10^6 ^*B. cereus *cells per reaction could be detected with no background noise [11]. Currently this technique is modifying to further improve the sensitivity and adapting it to detect other pathogenic microorganisms.

From another point of view, work with synthetic nucleic acid analogs is required when developing novel methods that allow to meet sensitivity and multiplexing needs. The main drawback of assays using PCR amplicons as targets is that they are not quantitative. Due to saturation of the amplification reaction, the amount of such targets provided for biochip analysis differs from the initial material level. The quantification may be improved by using a low cycle number PCR approach. By limiting the PCR cycle number only the exponential phase of amplification will be included. Thus, further development of electrical methods with focus on biochip techniques that can be coupled to low cycle number PCR, or even do not use PCR amplification at all is required.

As it is discussed above, the main limiting factor for the development of biochips can be the sensitivity. To achieve low detection limits reliably it is possible to increase the amount of target in the sample or to amplify the signal. Many of the detection systems do both at the same time. PCR, although with its several negative aspects such as being time consuming technique, is the most commonly used method to increase the amount of target in the sample. In order to minimize assay time, PCR has been integrated into a biochip system [48]. However, as alternative to the popular PCR and related amplification approaches, an additional trend is directed to nonamplification strategies such as multi-labeling, branched chain or dendrimer type assay.

The sensitive and discriminating detection of the hybridization event is an important feature. Parameters of the hybridization protocol, like salt-concentration in the hybridization buffer, hybridization temperature, addition of a helper probe, or a fragmentation of the nucleic acid prior to the hybridization, that are known from literature to influence the result of a hybridization reaction should be optimized. It was reported that the problems caused by the higher-order structure of nucleic acid target could be solve by the addition of unlabeled helper oligonucleotides that bind in close proximity to the probes. In this respect, the helpers have a synergistic effect for the probe binding [59,60]. To overcome the problems caused by higher-ordered structures of nucleic acid targets [55], especially during RNA work, the fragmentation of the nucleic acid is recommended. Such structures became a significant concern in the design and application of biochips. Application of ultrasound to provide nucleic acid fragments suitable for detection by nucleic acid chips was presented [45]. By the generation of smaller sized nucleic acid molecules a fragmentation of the nucleic acid could antagonize negative effects on the hybridization efficiency caused by steric hindrances.

## Conclusion

Biosensors are making a great impact on the development of rapid and sensitive assays for the detection of pathogenic microorganisms. It is assumed that nucleic acid chip arrays, nowadays broadly used in the area of biomolecule typing, in the close future may become an extremely important tool for pathogen detection. Although the present systems provide many advantages, there are still other matters to be addressed if considering their commercialization for diagnostics. Absolute automation of the sample handling and integration with the system is important. This will minimize problems due to contamination and avoid human error. In addition, further reduction in the analyzing time as well as steps forward better sensitivity should be considered. The scale-down of nucleic acid chip from the micro-scale to nano-scale will benefit over current technologies.

In general, high sensitivity and high selectivity using a protocol that can be completed in a relatively short time are requirements for an ideal detector nowadays. Although the optical types of biosensors are the most commonly employed at the moment, the electrical ones can be expected to give the lowest detection limits along with the most simple instrumentation for on-site and point-of-care applications.
